# Supplementary material for: Brightening triplet excitons enable high-performance white-light emission in organic small molecules via integrating n–π*/π–π* transitions
Source: Nat Commun. 2024 Sep 5;15:7778. doi: 10.1038/s41467-024-52196-7 (PMC11377787; doi:10.1038/s41467-024-52196-7)
Supplement: Supplementary file 1 — Supplementary Information [file 41467_2024_52196_MOESM1_ESM.pdf]

Supplementary Information
for
Brightening triplet excitons enable high-performance white-light emission in organic small molecules via integrating *n*-π*/π-π* transitions

Qing Yang[1], Xinyi Yang[1]*, Yixuan Wang[1], Yunfan Fei[2], Fang Li[2], Haiyan Zheng[2], Kuo Li[2], Yibo Han[3], Takanori Hattori[4], Pinwen Zhu[1], Shuaiqiang Zhao[5], Leiming Fang[6], Xuyuan Hou[1], Zhaodong Liu[1], Bing Yang[5] and Bo Zou[1]*

[1] *State Key Laboratory of Superhard Materials, Synergetic Extreme Condition High-Pressure Science Center, College of Physics, Jilin University, Changchun, 130012, China.*

[2] *Center for High Pressure Science and Technology Advanced Research, Beijing 100193, China.*

[3] *Wuhan National High Magnetic Field Center and School of Physics, Huazhong University of Science and Technology, Wuhan, Hubei 430074, China.*

[4] *J-PARC Center, Japan Atomic Energy Agency, Tokai, Ibaraki 319-1195, Japan.*

[5] *State Key Laboratory of Supramolecular Structure and Materials, College of Chemistry, Jilin University, Changchun, 130012, China.*

[6] *Institute of Nuclear Physics and Chemistry, China Academy of Engineering Physics, Mianyang 621900, China*

*Corresponding authors: yangxinyi@jlu.edu.cn (X.Y.); zoubo@jlu.edu.cn (B.Z.)

Supplementary Figures and Tables

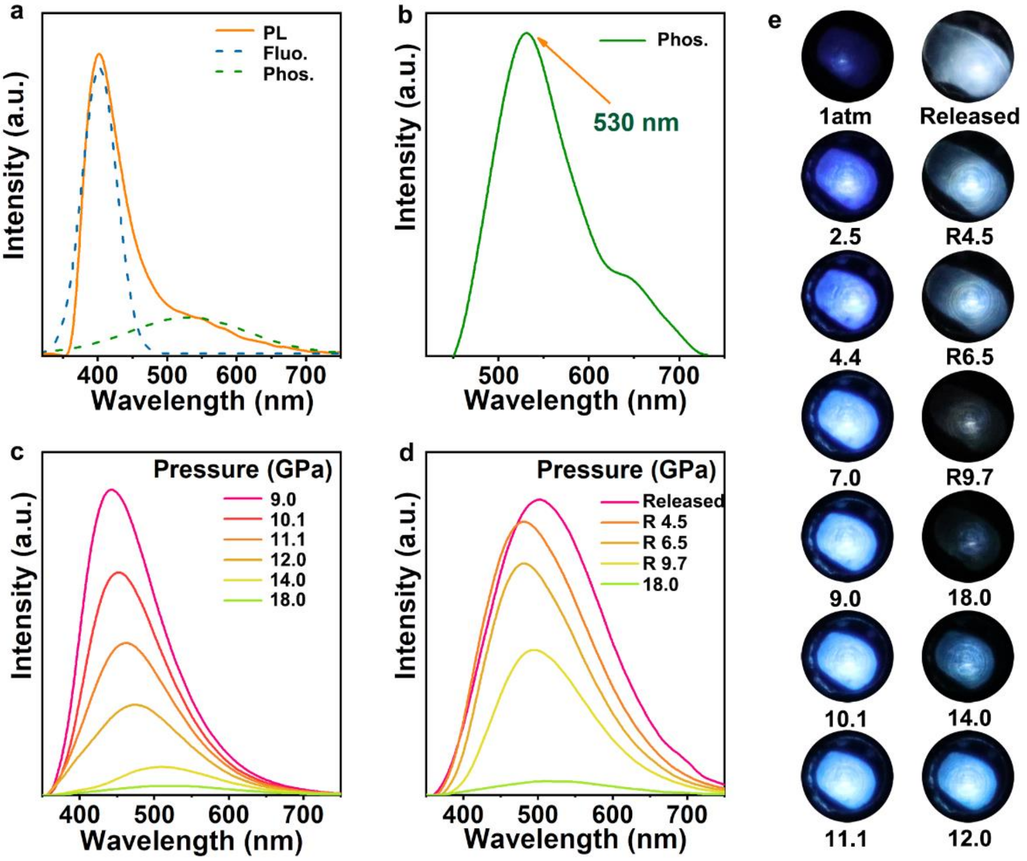

Supplementary Fig. 1 | PL evolution with PTM of silicone oil upon compression and decompression. **a** PL (orange solid line), fitting fluorescence (blue dashed line), and fitting phosphorescence (green dashed line) spectra of isophthalic acid (IPA) at ambient conditions. **b** Room-temperature phosphorescence spectrum of the pristine IPA at ambient conditions. **c** PL spectra of IPA upon compression to 18.0 GPa from 9.0 GPa. **d** PL spectra of IPA upon decompression. **e** The corresponding PL photographs upon compression and decompression.

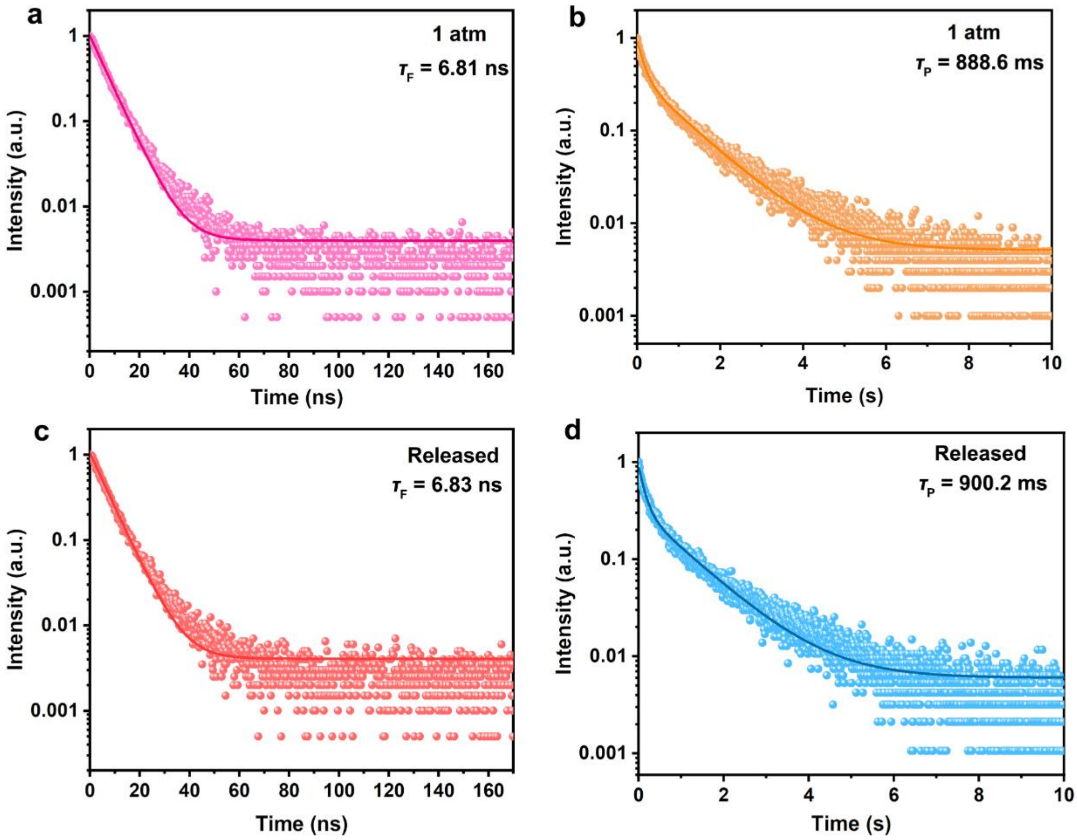

Supplementary Fig. 2 | The time-resolved PL decay curves. The time-resolved PL decay curves of the pristine IPA measured at **a** 402 nm and **b** 530 nm. The time-resolved PL decay curves of the pressure-treated IPA (treated by the Walker-Type Large-Volume Press upon decompression from 20 GPa) measured at **c** 481 nm and **d** 573 nm.

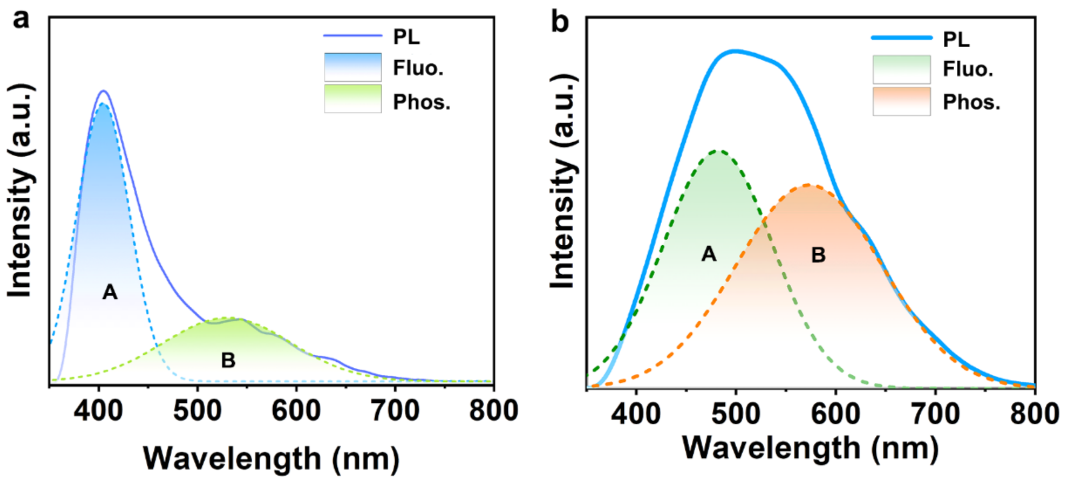

Supplementary Fig. 3 | Peak-differentiation-imitating analysis of the initial and pressure-treated PL spectra. Peak-differentiation-imitating analysis of fluorescence and phosphorescence parts of the **a** pristine and **b** treated IPA (treated by the Walker-Type Large-Volume Press upon decompression from 20 GPa). The fluorescence yield (Φ_F) is defined by: Φ_F=Φ*A/(A+B). The RTP yield (Φ_P) is defined by: Φ_P=Φ*B/(A+B). Φ is the total absolute photoluminescent quantum yields[1].

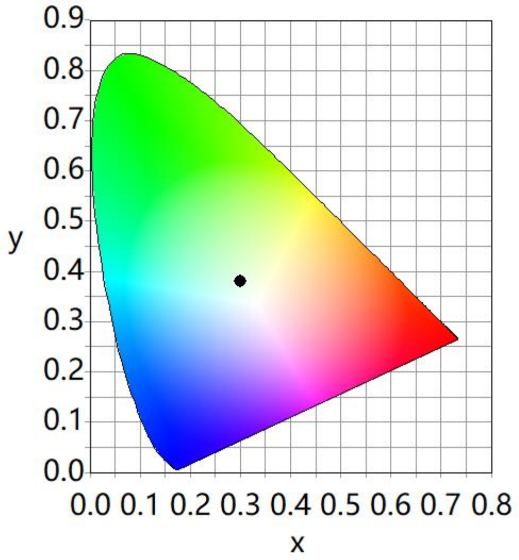

Supplementary Fig. 4 | Chromaticity coordinates. The chromaticity coordinates (0.30, 0.38) of the compressed IPA at 18.0 GPa.

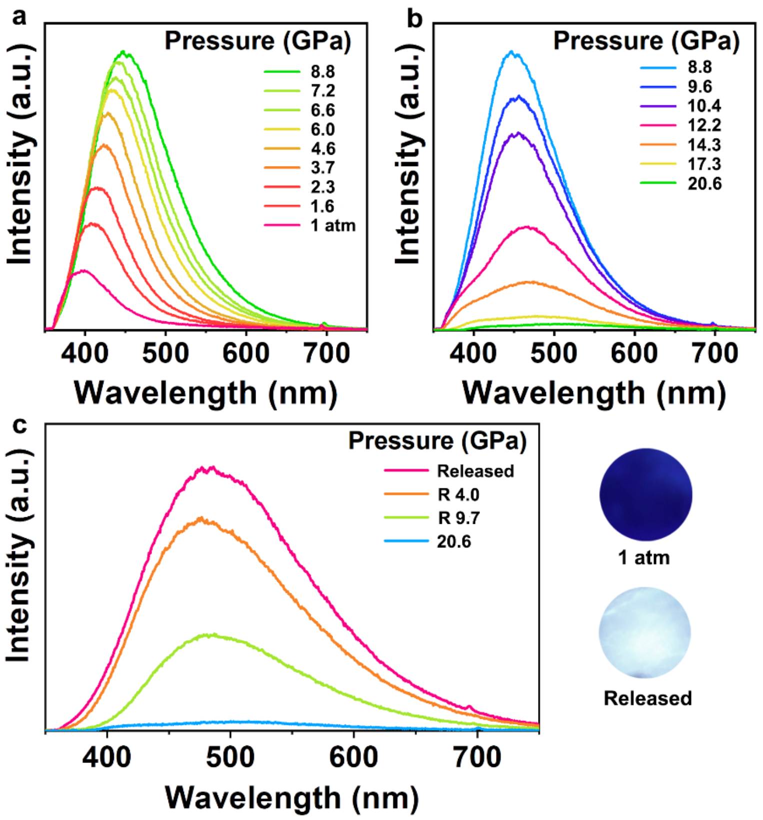

Supplementary Fig. 5 | PL evolution of IPA with PTM of liquid argon upon compression and decompression. a PL spectra of IPA with PTM of liquid argon upon compression to 8.8 GPa. **b** PL spectra of IPA upon compression from 8.8 GPa to 20.6 GPa. **c** PL spectra of IPA upon decompression. The right figure shows the corresponding PL photographs at ambient conditions and released from 20.6 GPa.

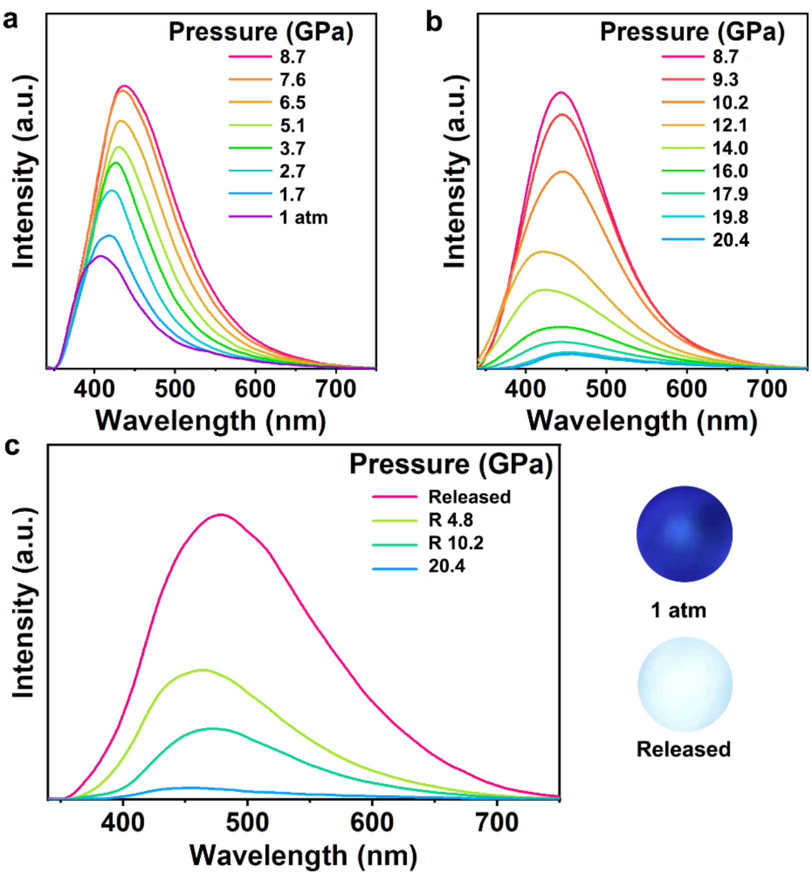

Supplementary Fig | 6. PL evolution of IPA with PTM of liquid nitrogen upon compression and decompression. a PL spectra of IPA with PTM of liquid nitrogen upon compression to 8.7 GPa. **b** PL spectra of IPA upon compression from 8.7 GPa to 20.4 GPa. **c** PL spectra of IPA upon decompression. The right figure shows the corresponding PL photographs at ambient conditions and released from 20.4 GPa.

In order to further investigate the influence of PTM on the PL properties of IPA, we supplemented *in-situ* high-pressure PL experiments by using liquid argon (Supplementary Fig. 5) and nitrogen (Supplementary Fig. 6) as PTM, respectively. We found that the PL evolution from these two experimental runs was basically consistent with that of silicone oil. After ~20.0 GPa of depressurization, we still harvest a bright white-light emission with liquid argon and nitrogen, respectively. Therefore, the different PTMs (silicone oil, liquid argon, and nitrogen) could not influence the variation trend of optical properties and the harvest of the resulting enhanced white-light emission.

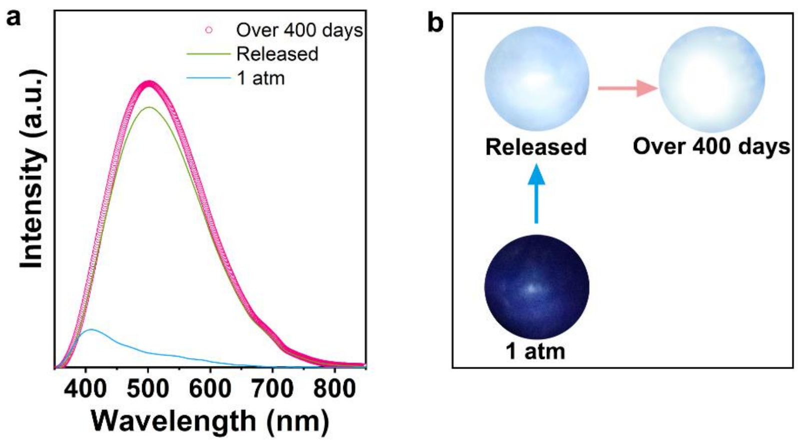

Supplementary Fig. 7 | PL stability analysis. a The PL spectra of IPA. **b** Optical images of IPA at 1 atm and upon complete release of the pressure. The pressure-treated samples were stored at ambient conditions. The corresponding temperature was about 18-25 °C, and the humidity was about 40 %-50 %.

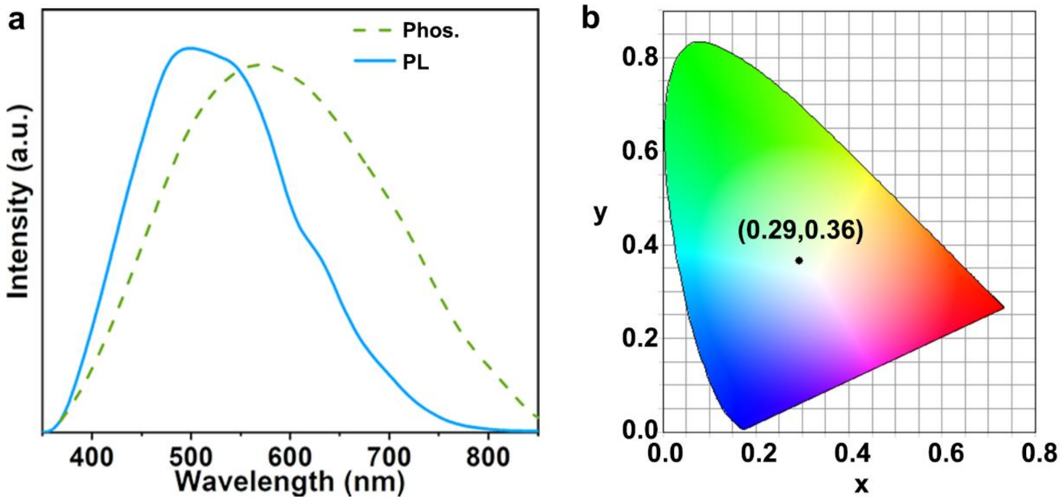

Supplementary Fig. 8 | Characterization of optical properties of pressure-treated IPA. a The normalized steady-state PL (blue solid line) and phosphorescence (green solid line) spectra for the treated sample after a compression cycle of 1 atm to 20.0 GPa using the Walker-Type Large-Volume Press. After turning off the laser excitation, the lower-energy band was measured located ~573 nm. **b** The chromaticity coordinates (0.29, 0.36) of the corresponding pressure-treated IPA. These results indicated that the white-light emission treated by Walker-Type Large-Volume Press was consistent with the result treated by DAC.

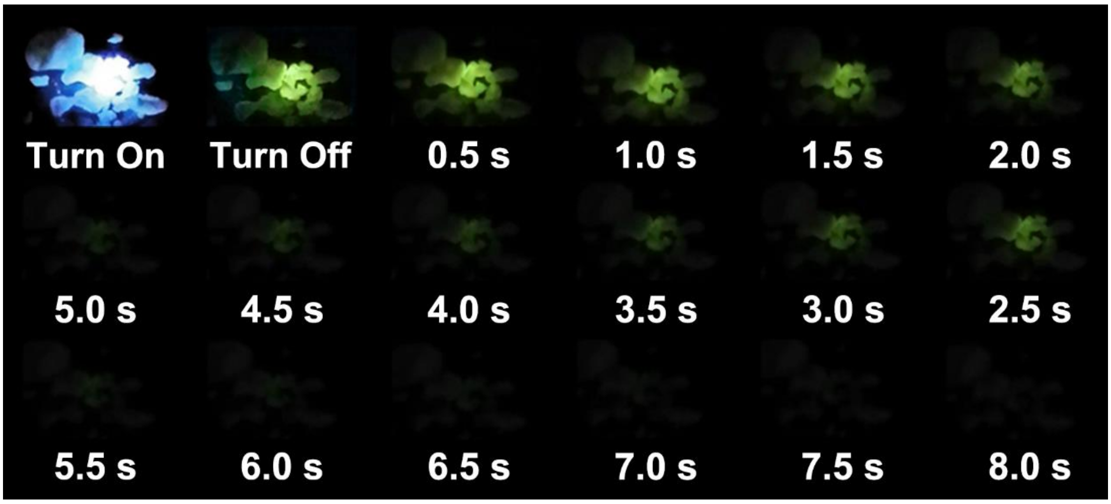

Supplementary Fig. 9 | Photographs of the phosphorescence. Photographs of the phosphorescence of the targeted IPA (treated by the Walker-Type Large-Volume Press) taken at different time intervals before and after turning off the UV excitation (355 nm) under ambient conditions.

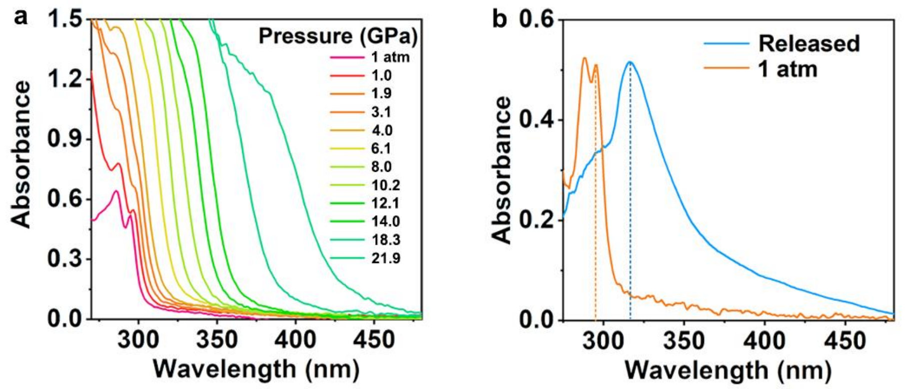

Supplementary Fig. 10 | UV-vis absorption evolution upon compression and decompression. a UV-vis absorption spectra of IPA upon compression. **b** UV-vis absorption spectra of IPA at ambient conditions and after pressure was completely released. The absorption peak at 1 atm was located at 295 nm (orange line), with an estimated bandgap of 4.20 eV. The absorption peak after completely releasing the pressure was located at 317 nm (blue line), with an estimated bandgap of 3.91 eV.

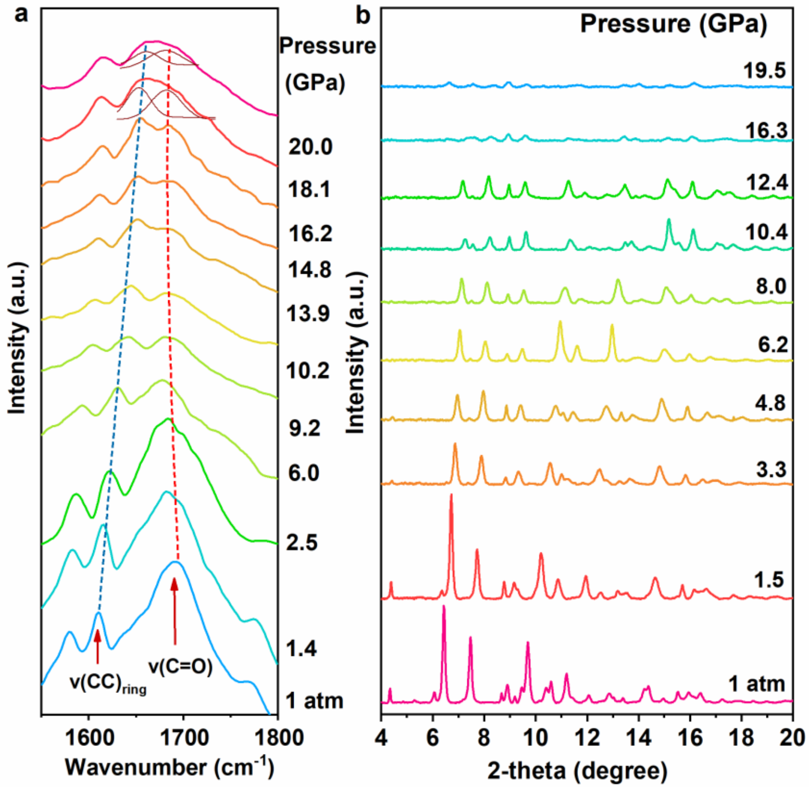

Supplementary Fig. 11 | Structural analysis of IPA upon compression and decompression. a Selected IR spectra of IPA in the region of C=O stretching vibrational mode (ν(C=O)) upon compression. **b** ADXRD patterns of the IPA crystal under different pressures (λ = 0.6199 Å).

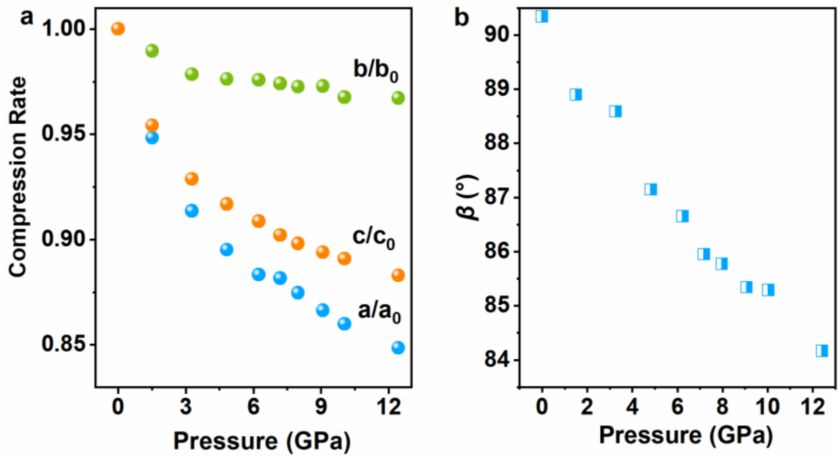

Supplementary Fig. 12 | Structural parameters evolution upon compression. a The compression rate of three lattice constants (a, b, c) at different pressures. **b** Pressure-dependent β evolution of IPA. These parameters were determined by Rietveld refinement of ADXRD patterns.

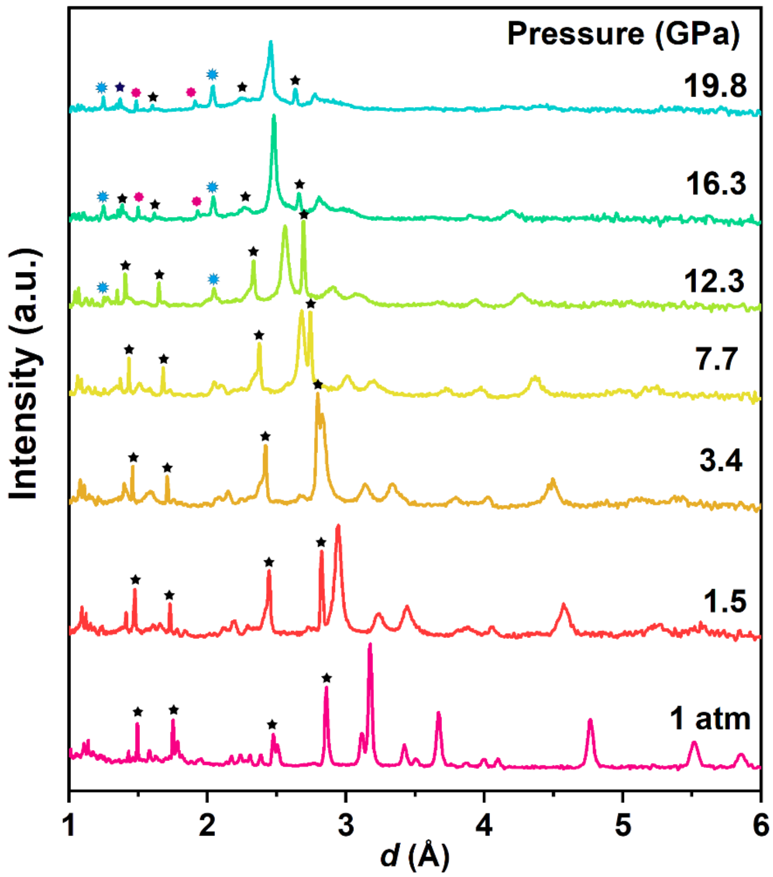

Supplementary Fig. 13 | High-pressure neutron spectra. Time-of-flight (TOF) neutron diffraction patterns of IPA-d_6 upon compression. The black asterisks indicate the peaks of the Pb marker. The magenta asterisks indicate the peaks of the Ar[2]. The blue asterisks indicate the peaks of the diamond. The pressure was estimated based on the EOS of Pb.

Synthesis of isophthalic acid-d_6: The deuterated IPA was prepared according to the literatures[3,4] In a Teflon vessel, platinum (IV) oxide (22.7 mg, 0.1 mmol), deuterium oxide (15 mL), and IPA (2.0 mmol) were added, then the vessel was placed in autoclave and sealed. The autoclave was heated to 250 °C in an oven and cooled to room temperature after 24 h. Platinum (IV) oxide was removed by filtration, and the filtrate was concentrated by rotary evaporation. The crude product was dissolved in 100 ml acetone-d_6 and the solvent was carefully dried over anhydrous Na_2SO_4. After filtration and concentration under vacuum, the pure product was obtained.

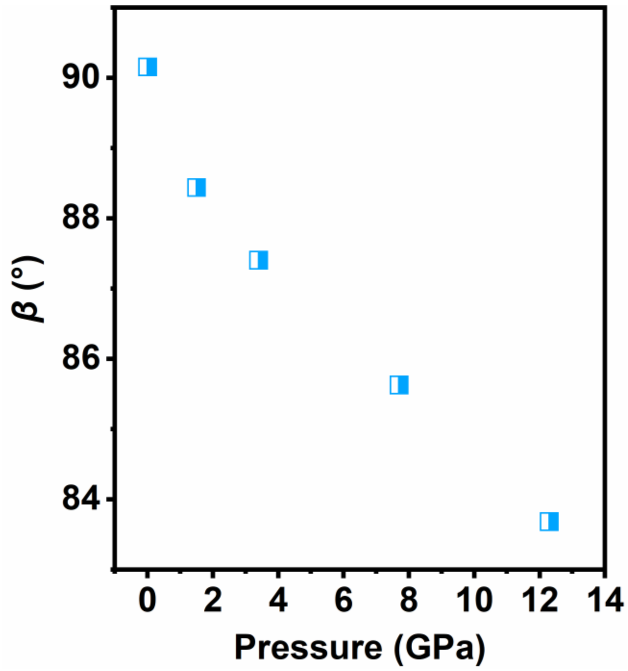

Supplementary Fig. 14 | Structural parameters evolution upon compression. Pressure-dependent β evolution of IPA. The parameter was determined by Rietveld refinement of neutron diffraction patterns.

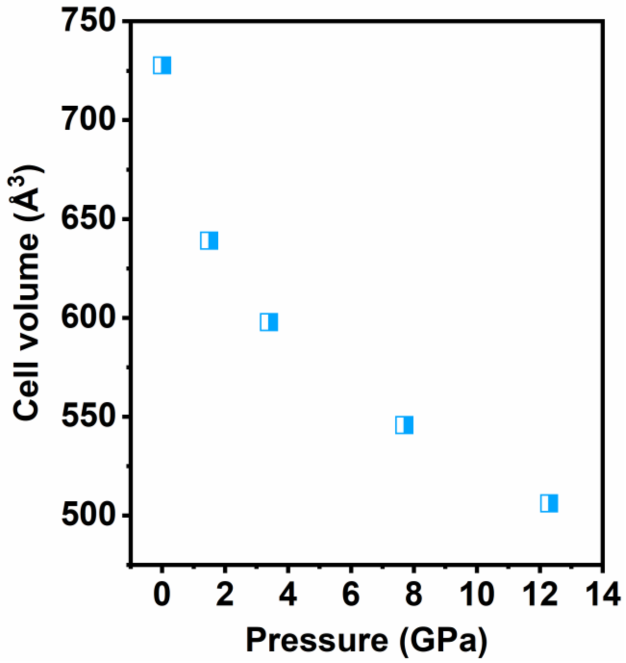

Supplementary Fig. 15 | Structural parameters evolution upon compression. Cell volume evolutions of IPA upon compression.

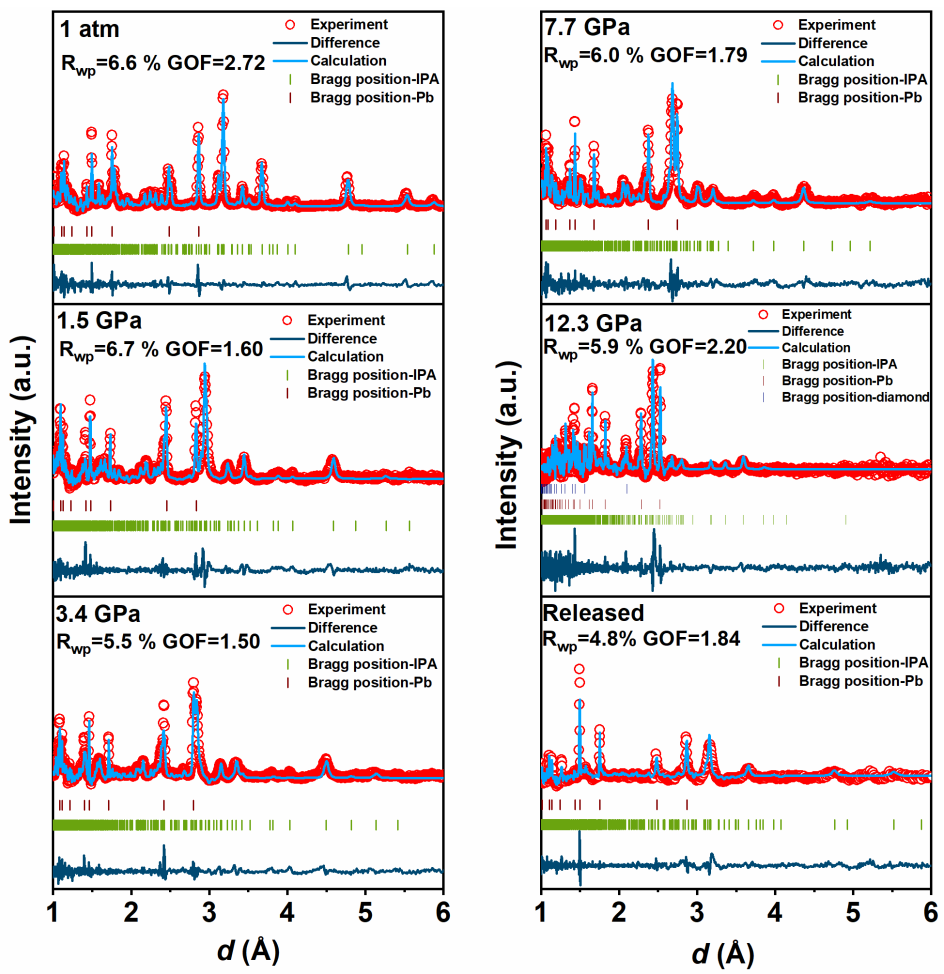

Supplementary Fig. 16 | Rietveld refinement. Rietveld refinement plot of IPA-d_6 upon compression and decompression. The experimental data was collected at BL11 PLANET of the J-PARC. Before the high-pressure experiment, the fresh sample with a Pb marker was loaded into a vanadium can to collect the neutron diffraction data. The data were used as an ambient-pressure reference. The high-pressure neutron diffraction patterns were collected by loading the sample with Pb and Ar in VX4 Paris-Edinburgh (PE) Press.

Based on the *in situ* TOF neutron diffraction experiments, we further performed Rietveld refinements of neutron diffraction patterns to obtain detailed lattice parameters and other structural information upon compression and decompression. With increasing pressure, lattice parameters *a*, *b*, and *c* experienced a decrease (Fig. 2c), and *β* underwent continuous reduction throughout the compression process (Supplementary Fig. 14). When refining the atom positions, we added restrictions on the bond length and bond angle of IPA to keep their variation within a reasonable range and avoid

molecular disintegration. In this regard, we conducted the evolution of the intermolecular hydrogen bond distances $D_{17}\cdots O_9$ (d_1), $D_{21}\cdots O_5$ (d_2) and the parallel misalignment angle (σ) upon compression (Fig. 2e, f).

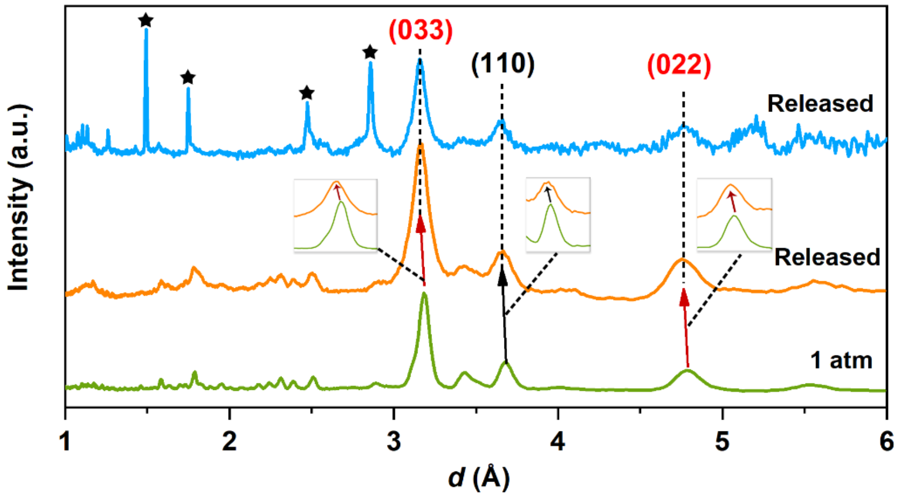

Supplementary Fig. 17 | The neutron diffraction patterns. Neutron diffraction patterns of IPA-d_6 before and after pressure treatment. The neutron diffraction patterns of the pristine (green line) and recovered (orange line) samples were collected at High pressure neutron diffractometer (Fenghuang) at China Mianyang Research Reactor's (CMRR) neutron science platform. The neutron diffraction pattern of the recovered (blue line) sample was collected at BL11 PLANET in the Materials and Life Science Experimental Facility (MLF) at Japan Proton Accelerator Research Complex (J-PARC). The black asterisks indicate the peaks of the Pb marker.

We conducted further experiments on the recovered sample after removing the gasket. This neutron diffraction data for the pristine and recovered sample (removing the gasket) were collected at High pressure neutron diffractometer (Fenghuang) at China Mianyang Research Reactor's (CMRR) neutron science platform.

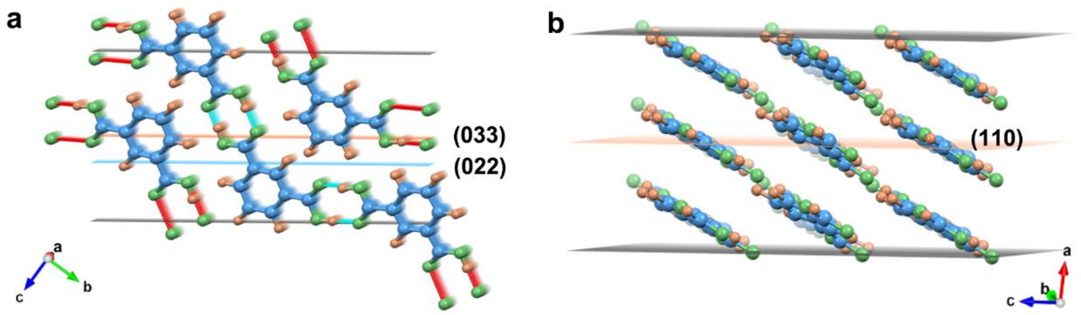

Supplementary Fig. 18 | The lattice planes of IPA. a The lattice planes of (033) and (022) in IPA. **b** The lattice planes of (110) in IPA.

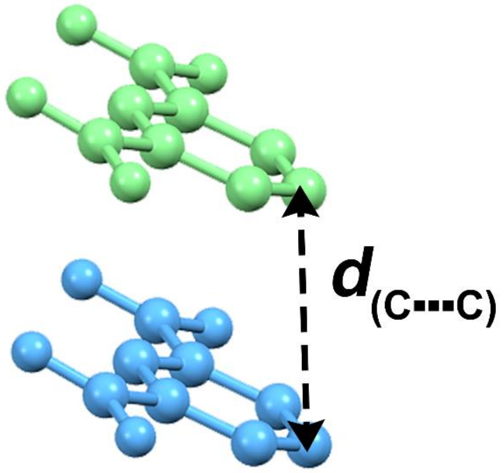

Supplementary Fig. 19 | The crystal structure of IPA. The black arrows indicate the C···C distance.

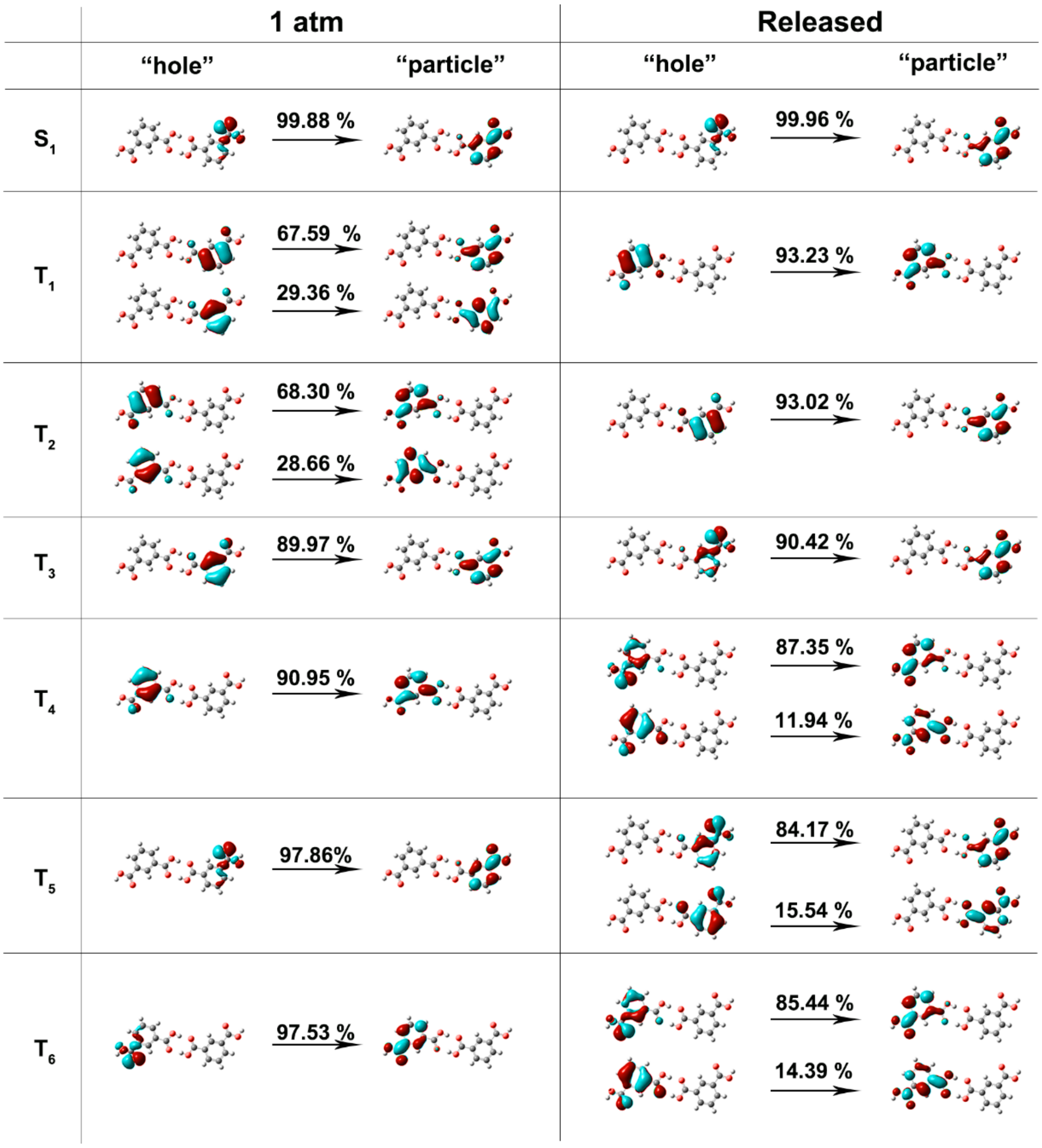

Supplementary Fig. 20 | The NTOs of IPA. NTOs of S_1 and T_n ($1 \leq n \leq 6$) states of the pristine (1 atm) and pressure-treated IPA.

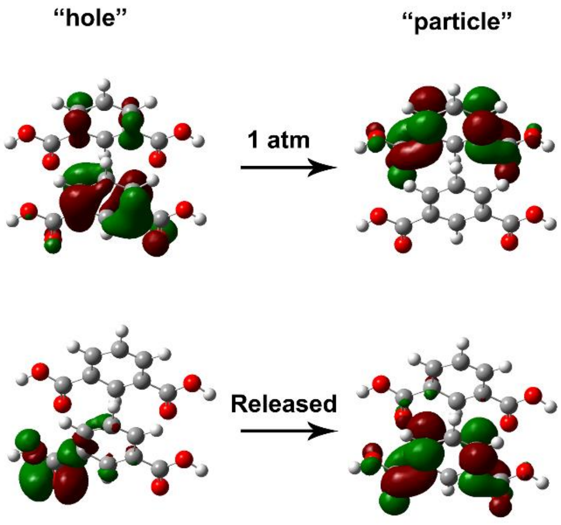

Supplementary Fig.21 | The molecular orbitals of IPA. The molecular orbitals for the π-π staking dimers in the pristine and pressure-treated IPA.

Supplementary Table 1 | Photophysical data of IPA upon compression and decompression.

Pressure (GPa)	PLQY (%)	Φ_F (%)	Φ_P (%)	τ_F (ns)	τ_P (s)	K_F (ns^{-1})	K_{ISC} (ns^{-1})	K_P (s^{-1})	K_{nr} (s^{-1})	K_{nr}' (s^{-1})
1 atm	19	12	7	6.81	0.8886	0.018	0.010	0.079	1.047	0.119
Released	75	35	40	6.83	0.9002	0.051	0.059	0.444	0.667	0.037

The radiative decay rate constant of fluorescence $K_F = \Phi_F/\tau_F$. The nonradiative decay rate constant of fluorescence $K_{nr}' = (1-\Phi_F - \Phi_P)/\tau_F$. The intersystem crossing rate constant $K_{ISC} = \Phi_P/\tau_F$. The radiative decay rate constant of phosphorescence $K_P = \Phi_P/\tau_P$. The nonradiative decay rate constant of phosphorescence $K_{nr} = (1-\Phi_P)/\tau_P$[5].

Supplementary Table 2 | Lattice parameters of IPA upon compression and decompression. These values were determined by Rietveld refinement using the GSASII software package based on neutron diffraction data.

Pressure (GPa)	*a* (Å)	*b* (Å)	*c* (Å)	*β* (°)	*α*=*γ* (°)
1 atm	3.770	16.404	11.766	90.155	90.00
1.5	3.528	16.268	11.136	88.439	90.00
3.4	3.421	16.128	10.847	87.404	90.00
7.7	3.283	15.931	10.466	85.631	90.00
12.3	3.164	15.716	10.2406	83.685	90.00
Released	3.755	16.283	11.741	89.800	90.00

Supplementary Table 3 | The d_1, d_2, σ, and $d_{(C\cdots C)}$ values of IPA upon compression and decompression.

**Pressure (GPa)**	d_1	d_2	σ (°)	$d_{(C\cdots C)}$
1 atm	1.73(4)	1.77(4)	67.7(8)	3.77(5)
1.5	1.67(4)	1.71(5)	63.5(7)	3.53(4)
3.4	1.49(3)	1.54(3)	62.4(7)	3.42(4)
7.7	1.45(3)	1.48(3)	62.0(6)	3.28(3)
12.3	1.37(4)	1.40(5)	60.7(9)	3.16(4)
Released	1.49(5)	1.69(5)	65.7(9)	3.75(5)

Supplementary Table 4 | The SOC constants (ξ) of the pristine and pressure-treated IPA.

$\xi(S_1\text{-}T_n)$	$\xi(S_1\text{-}T_1)$ (cm^{-1})	$\xi(S_1\text{-}T_2)$ (cm^{-1})	$\xi(S_1\text{-}T_3)$ (cm^{-1})	$\xi(S_1\text{-}T_4)$ (cm^{-1})	$\xi(S_1\text{-}T_5)$ (cm^{-1})	$\xi(S_1\text{-}T_6)$ (cm^{-1})
1 atm	0.73	5.42	13.64	5.27	28.53	0.08
Released	1.92	19.35	18.55	11.34	29.18	7.71

Supplementary Table 5 | The energies of singlet and triplet states of the pristine and pressure-treated IPA.

Energy	S_1 (eV)	T_1 (eV)	T_2 (eV)	T_3 (eV)	T_4 (eV)	T_5 (eV)	T_6 (eV)
1 atm	4.62	3.44	3.48	3.65	3.72	3.83	3.96
Released	3.59	2.39	2.54	3.17	3.31	3.46	3.51

Supplementary Table 6 | The $\Delta E(S_1\text{-}T_n)$ of the pristine and pressure-treated IPA.

$\Delta E(S_1\text{-}T_n)$	$\Delta E(S_1\text{-}T_1)$ (eV)	$\Delta E(S_1\text{-}T_2)$ (eV)	$\Delta E(S_1\text{-}T_3)$ (eV)	$\Delta E(S_1\text{-}T_4)$ (eV)	$\Delta E(S_1\text{-}T_5)$ (eV)	$\Delta E(S_1\text{-}T_6)$ (eV)
1 atm	1.18	1.14	0.97	0.90	0.79	0.66
Released	1.20	1.05	0.42	0.28	0.13	0.08

Crystal structure of IPA at different pressures.

1 atm

```
data_1 atm
_gsas_GSASII_version    5455

# phase info for 0 follows
_pd_phase_name    0
_cell_measurement_temperature    ?
_cell_length_a    3.76987
_cell_length_b    16.40422
_cell_length_c    11.76636
_cell_angle_alpha    90
_cell_angle_beta    90.155
_cell_angle_gamma    90
_cell_volume    727.65
_exptl_crystal_density_diffrn    1.5716
_symmetry_cell_setting    monoclinic
_symmetry_space_group_name_H-M    "P 21/c"
loop_
    _space_group_symop_id
    _space_group_symop_operation_xyz
     1    x,y,z
     2    -x,1/2+y,1/2-z
     3    -x,-y,-z
     4    x,1/2-y,1/2+z

# ATOMIC COORDINATES AND DISPLACEMENT PARAMETERS
loop_
   _atom_site_label
   _atom_site_type_symbol
   _atom_site_fract_x
   _atom_site_fract_y
   _atom_site_fract_z
   _atom_site_occupancy
   _atom_site_adp_type
   _atom_site_U_iso_or_equiv
   _atom_site_site_symmetry_multiplicity
C1     C    0.731(8)     0.3440(11)   0.3724(20)   1.0000    Uiso 0.0127   4
C5     C    0.690(8)     0.2697(11)   0.3189(19)   1.0000    Uiso 0.0127   4
C9     C    0.769(8)     0.2033(12)   0.3868(19)   1.0000    Uiso 0.0127   4
C13    C    0.890(9)     0.2075(12)   0.5058(19)   1.0000    Uiso 0.0127   4
C17    C    -0.009(9)    0.2822(11)   0.5518(19)   1.0000    Uiso 0.0127   4
```

C21	C	0.933(10)	0.3501(13)	0.4814(19)	1.0000	Uiso 0.0127	4
C25	C	0.653(8)	0.4203(12)	0.3067(23)	1.0000	Uiso 0.0127	4
C29	C	0.639(9)	0.1236(12)	0.3365(23)	1.0000	Uiso 0.0127	4
H1	H	0.540(8)	0.2604(16)	0.2342(24)	1.0000	Uiso 0.0127	4
H5	H	-0.013(9)	0.1471(16)	0.5478(22)	1.0000	Uiso 0.0127	4
H9	H	0.129(8)	0.2891(16)	0.6357(23)	1.0000	Uiso 0.0127	4
H13	H	-0.039(8)	0.4111(15)	0.5244(23)	1.0000	Uiso 0.0127	4
H17	H	0.676(10)	0.5317(18)	0.3126(26)	1.0000	Uiso 0.0127	4
H21	H	0.612(10)	0.0088(17)	0.3630(25)	1.0000	Uiso 0.0127	4
O1	O	0.752(8)	0.4886(18)	0.3622(23)	1.0000	Uiso 0.0127	4
O5	O	0.493(11)	0.4204(17)	0.2166(29)	1.0000	Uiso 0.0127	4
O9	O	0.473(11)	0.1228(17)	0.244(3)	1.0000	Uiso 0.0127	4
O13	O	0.698(9)	0.0534(18)	0.4022(26)	1.0000	Uiso 0.0127	4

```
loop_    _atom_type_symbol _atom_type_number_in_cell
   C           32
   H           24
   O           16

# Note that Z affects _cell_formula_sum and _weight
_cell_formula_units_Z    4
_chemical_formula_sum    "C8 H6 O4"
_chemical_formula_weight    172.17
```

1.5 GPa

```
data_1.5 GPa
_gsas_GSASII_version    5455

# phase info for 1 follows
_pd_phase_name    1
_cell_measurement_temperature    ?
_cell_length_a    3.52815
_cell_length_b    16.26764
_cell_length_c    11.13634
_cell_angle_alpha    90
_cell_angle_beta      88.439
_cell_angle_gamma    90
_cell_volume    638.929
_exptl_crystal_density_diffrn    1.7898
_symmetry_cell_setting    monoclinic
_symmetry_space_group_name_H-M    "P 21/c"
loop_
        _space_group_symop_id
        _space_group_symop_operation_xyz
          1    x,y,z
          2    -x,1/2+y,1/2-z
          3    -x,-y,-z
          4    x,1/2-y,1/2+z

# ATOMIC COORDINATES AND DISPLACEMENT PARAMETERS
loop_
      _atom_site_label
      _atom_site_type_symbol
      _atom_site_fract_x
      _atom_site_fract_y
      _atom_site_fract_z
      _atom_site_occupancy
      _atom_site_adp_type
      _atom_site_U_iso_or_equiv
      _atom_site_site_symmetry_multiplicity
C1     C    0.747(10)    0.3451(9)     0.3726(21)    1.0000    Uiso 0.0127    4
C5     C    0.680(9)     0.2676(9)     0.3265(19)    1.0000    Uiso 0.0127    4
C9     C    0.751(11)    0.1979(10)    0.3949(23)    1.0000    Uiso 0.0127    4
C13    C    0.930(10)    0.2029(11)    0.5047(20)    1.0000    Uiso 0.0127    4
C17    C    0.013(8)     0.2798(11)    0.5493(19)    1.0000    Uiso 0.0127    4
C21    C    0.894(10)    0.3515(11)    0.4891(21)    1.0000    Uiso 0.0127    4
C25    C    0.653(12)    0.4212(10)    0.3044(24)    1.0000    Uiso 0.0127    4
C29    C    0.637(10)    0.1182(11)    0.3441(23)    1.0000    Uiso 0.0127    4
```

H1	H	0.538(9)	0.2610(16)	0.2447(26)	1.0000	Uiso 0.0127	4
H5	H	-0.047(8)	0.1477(15)	0.5600(24)	1.0000	Uiso 0.0127	4
H9	H	0.131(10)	0.2840(17)	0.6379(23)	1.0000	Uiso 0.0127	4
H13	H	0.004(12)	0.4140(15)	0.5155(29)	1.0000	Uiso 0.0127	4
H17	H	0.641(10)	0.5352(16)	0.3205(30)	1.0000	Uiso 0.0127	4
H21	H	0.648(11)	0.0060(25)	0.3616(30)	1.0000	Uiso 0.0127	4
O1	O	0.726(10)	0.4889(15)	0.3645(25)	1.0000	Uiso 0.0127	4
O5	O	0.522(13)	0.4210(18)	0.2091(28)	1.0000	Uiso 0.0127	4
O9	O	0.505(11)	0.1186(19)	0.2523(30)	1.0000	Uiso 0.0127	4
O13	O	0.744(12)	0.0496(16)	0.399(4)	1.0000	Uiso 0.0127	4

loop_ _atom_type_symbol _atom_type_number_in_cell

C	32
H	24
O	16

Note that Z affects _cell_formula_sum and _weight
_cell_formula_units_Z 4
_chemical_formula_sum "C8 H6 O4"
_chemical_formula_weight 172.17

3.4 GPa

```
data_3.4 GPa
_gsas_GSASII_version    5455

# phase info for 3 follows
_pd_phase_name    3
_cell_measurement_temperature    ?
_cell_length_a    3.42059
_cell_length_b    16.1281
_cell_length_c    10.84673
_cell_angle_alpha    90
_cell_angle_beta      87.404
_cell_angle_gamma    90
_cell_volume    597.774
_exptl_crystal_density_diffrn    1.9130
_symmetry_cell_setting    monoclinic
_symmetry_space_group_name_H-M    "P 21/c"
loop_
    _space_group_symop_id
    _space_group_symop_operation_xyz
     1    x,y,z
     2    -x,1/2+y,1/2-z
     3    -x,-y,-z
     4    x,1/2-y,1/2+z

# ATOMIC COORDINATES AND DISPLACEMENT PARAMETERS
loop_
   _atom_site_label
   _atom_site_type_symbol
   _atom_site_fract_x
   _atom_site_fract_y
   _atom_site_fract_z
   _atom_site_occupancy
   _atom_site_adp_type
   _atom_site_U_iso_or_equiv
   _atom_site_site_symmetry_multiplicity
C1     C    0.713(9)    0.3447(8)    0.3857(19)    1.0000    Uiso 0.0127    4
C5     C    0.659(8)    0.2684(9)    0.3355(17)    1.0000    Uiso 0.0127    4
C9     C    0.731(8)    0.1949(8)    0.3986(18)    1.0000    Uiso 0.0127    4
C13    C    0.913(8)    0.2000(9)    0.5077(18)    1.0000    Uiso 0.0127    4
C17    C    0.994(8)    0.2759(8)    0.5569(17)    1.0000    Uiso 0.0127    4
C21    C    0.886(9)    0.3482(9)    0.4984(18)    1.0000    Uiso 0.0127    4
C25    C    0.660(9)    0.4186(9)    0.3087(21)    1.0000    Uiso 0.0127    4
C29    C    0.651(9)    0.1150(9)    0.3405(19)    1.0000    Uiso 0.0127    4
```

H1	H	0.527(9)	0.2684(16)	0.2505(21)	1.0000	Uiso 0.0127	4
H5	H	0.927(11)	0.1451(13)	0.5601(22)	1.0000	Uiso 0.0127	4
H9	H	0.160(8)	0.2789(14)	0.6389(20)	1.0000	Uiso 0.0127	4
H13	H	0.980(9)	0.4059(13)	0.5302(23)	1.0000	Uiso 0.0127	4
H17	H	0.631(10)	0.5408(15)	0.3261(29)	1.0000	Uiso 0.0127	4
H21	H	0.658(10)	-0.0053(16)	0.3535(26)	1.0000	Uiso 0.0127	4
O1	O	0.760(10)	0.4862(14)	0.3614(22)	1.0000	Uiso 0.0127	4
O5	O	0.469(11)	0.4148(17)	0.2119(25)	1.0000	Uiso 0.0127	4
O9	O	0.498(10)	0.1166(16)	0.2385(24)	1.0000	Uiso 0.0127	4
O13	O	0.738(12)	0.0483(14)	0.3986(28)	1.0000	Uiso 0.0127	4

```
loop_    _atom_type_symbol _atom_type_number_in_cell
   C          32
   H          24
   O          16

# Note that Z affects _cell_formula_sum and _weight
_cell_formula_units_Z    4
_chemical_formula_sum    "C8 H6 O4"
_chemical_formula_weight    172.17
```

7.7 GPa

```
data_7.7 GPa
_gsas_GSASII_version    5455

# phase info for 7 follows
_pd_phase_name    7
_cell_measurement_temperature    ?
_cell_length_a    3.28269
_cell_length_b    15.93102
_cell_length_c    10.46593
_cell_angle_alpha    90
_cell_angle_beta      85.631
_cell_angle_gamma    90
_cell_volume    545.742
_exptl_crystal_density_diffrn    2.0954
_symmetry_cell_setting    monoclinic
_symmetry_space_group_name_H-M    "P 21/c"
loop_
        _space_group_symop_id
        _space_group_symop_operation_xyz
          1    x,y,z
          2    -x,1/2+y,1/2-z
          3    -x,-y,-z
          4    x,1/2-y,1/2+z

# ATOMIC COORDINATES AND DISPLACEMENT PARAMETERS
loop_
      _atom_site_label
      _atom_site_type_symbol
      _atom_site_fract_x
      _atom_site_fract_y
      _atom_site_fract_z
      _atom_site_occupancy
      _atom_site_adp_type
      _atom_site_U_iso_or_equiv
      _atom_site_site_symmetry_multiplicity
C1     C    0.781(8)    0.3463(8)    0.8762(16)    1.0000    Uiso 0.0127    4
C5     C    0.697(7)    0.2665(8)    0.8292(17)    1.0000    Uiso 0.0127    4
C9     C    0.768(8)    0.1969(9)    0.9089(15)    1.0000    Uiso 0.0127    4
C13    C    0.969(7)    0.2047(9)    1.0243(16)    1.0000    Uiso 0.0127    4
C17    C    0.048(7)    0.2845(9)    1.0703(16)    1.0000    Uiso 0.0127    4
C21    C    0.926(7)    0.3539(9)    1.0035(16)    1.0000    Uiso 0.0127    4
C25    C    0.643(7)    0.4212(8)    0.8082(16)    1.0000    Uiso 0.0127    4
C29    C    0.689(7)    0.1144(9)    0.8554(16)    1.0000    Uiso 0.0127    4
```

H1	H	0.547(8)	0.2587(12)	0.7378(23)	1.0000	Uiso 0.0127	4
H5	H	0.039(7)	0.1498(12)	1.0797(20)	1.0000	Uiso 0.0127	4
H9	H	0.196(8)	0.2962(12)	1.1611(20)	1.0000	Uiso 0.0127	4
H13	H	0.988(7)	0.4180(12)	1.0374(19)	1.0000	Uiso 0.0127	4
H17	H	0.618(6)	0.5384(11)	0.8155(21)	1.0000	Uiso 0.0127	4
H21	H	0.652(7)	0.9930(13)	0.8713(25)	1.0000	Uiso 0.0127	4
O1	O	0.728(8)	0.4887(12)	0.8684(19)	1.0000	Uiso 0.0127	4
O5	O	0.465(8)	0.4208(14)	0.7011(22)	1.0000	Uiso 0.0127	4
O9	O	0.508(7)	0.1143(11)	0.7520(21)	1.0000	Uiso 0.0127	4
O13	O	0.785(8)	0.0448(12)	0.9160(21)	1.0000	Uiso 0.0127	4

```
loop_   _atom_type_symbol _atom_type_number_in_cell
   C          32
   H          24
   O          16

# Note that Z affects _cell_formula_sum and _weight
_cell_formula_units_Z    4
_chemical_formula_sum    "C8 H6 O4"
_chemical_formula_weight    172.17
```

12.3 GPa

```
data_12.3 GPa
_gsas_GSASII_version    5455

# phase info for 12.42p21c follows
_pd_phase_name    12.42p21c
_cell_measurement_temperature    ?
_cell_length_a    3.16379
_cell_length_b    15.71613
_cell_length_c    10.24018
_cell_angle_alpha    90
_cell_angle_beta     83.685
_cell_angle_gamma    90
_cell_volume    506.078
_exptl_crystal_density_diffrn    2.2597
_symmetry_cell_setting    monoclinic
_symmetry_space_group_name_H-M    "P 21/c"
loop_
    _space_group_symop_id
    _space_group_symop_operation_xyz
     1    x,y,z
     2    -x,1/2+y,1/2-z
     3    -x,-y,-z
     4    x,1/2-y,1/2+z

# ATOMIC COORDINATES AND DISPLACEMENT PARAMETERS
loop_
   _atom_site_label
   _atom_site_type_symbol
   _atom_site_fract_x
   _atom_site_fract_y
   _atom_site_fract_z
   _atom_site_occupancy
   _atom_site_adp_type
   _atom_site_U_iso_or_equiv
   _atom_site_site_symmetry_multiplicity
C1     C    0.747(14)    0.3482(11)    0.8784(27)    1.0000    Uiso 0.0127    4
C5     C    0.706(11)    0.2672(12)    0.8300(25)    1.0000    Uiso 0.0127    4
C9     C    0.761(12)    0.1988(12)    0.9119(26)    1.0000    Uiso 0.0127    4
C13    C    0.961(12)    0.2086(14)    1.0287(28)    1.0000    Uiso 0.0127    4
C17    C    0.055(10)    0.2894(12)    1.0675(25)    1.0000    Uiso 0.0127    4
C21    C    0.909(13)    0.3589(12)    0.9997(25)    1.0000    Uiso 0.0127    4
C25    C    0.642(13)    0.4227(12)    0.8036(31)    1.0000    Uiso 0.0127    4
C29    C    0.658(13)    0.1158(12)    0.8595(28)    1.0000    Uiso 0.0127    4
```

H1	H	0.540(14)	0.2593(19)	0.741(3)	1.0000	Uiso 0.0127	4
H5	H	0.087(14)	0.1551(19)	1.078(4)	1.0000	Uiso 0.0127	4
H9	H	0.147(12)	0.2997(20)	1.1638(29)	1.0000	Uiso 0.0127	4
H13	H	0.972(13)	0.4225(17)	1.028(3)	1.0000	Uiso 0.0127	4
H17	H	0.605(10)	0.5450(18)	0.8162(28)	1.0000	Uiso 0.0127	4
H21	H	0.669(13)	0.9915(20)	0.870(4)	1.0000	Uiso 0.0127	4
O1	O	0.727(12)	0.4931(18)	0.8645(28)	1.0000	Uiso 0.0127	4
O5	O	0.454(15)	0.4229(22)	0.704(4)	1.0000	Uiso 0.0127	4
O9	O	0.501(14)	0.1164(18)	0.750(3)	1.0000	Uiso 0.0127	4
O13	O	0.768(12)	0.0462(17)	0.918(3)	1.0000	Uiso 0.0127	4

```
loop_   _atom_type_symbol _atom_type_number_in_cell
   C          32
   H          24
   O          16

# Note that Z affects _cell_formula_sum and _weight
_cell_formula_units_Z    4
_chemical_formula_sum    "C8 H6 O4"
_chemical_formula_weight    172.17
```

Released

```
data_Released
_gsas_GSASII_version   5455

# phase info for 0 follows
_pd_phase_name   0
_cell_measurement_temperature   ?
_cell_length_a   3.75499
_cell_length_b   16.28341
_cell_length_c   11.74087
_cell_angle_alpha   90
_cell_angle_beta    89.8
_cell_angle_gamma   90
_cell_volume   717.88
_exptl_crystal_density_diffrn   1.5930
_symmetry_cell_setting   monoclinic
_symmetry_space_group_name_H-M   "P 21/c"
loop_
    _space_group_symop_id
    _space_group_symop_operation_xyz
     1   x,y,z
     2   -x,1/2+y,1/2-z
     3   -x,-y,-z
     4   x,1/2-y,1/2+z

# ATOMIC COORDINATES AND DISPLACEMENT PARAMETERS
loop_
   _atom_site_label
   _atom_site_type_symbol
   _atom_site_fract_x
   _atom_site_fract_y
   _atom_site_fract_z
   _atom_site_occupancy
   _atom_site_adp_type
   _atom_site_U_iso_or_equiv
   _atom_site_site_symmetry_multiplicity
C1    C    0.721(11)    0.3430(10)    0.8774(25)    1.0000    Uiso 0.0127    4
C5    C    0.652(12)    0.2667(11)    0.8267(24)    1.0000    Uiso 0.0127    4
C9    C    0.773(11)    0.1961(10)    0.8830(24)    1.0000    Uiso 0.0127    4
C13   C    0.909(14)    0.2007(11)    0.9978(27)    1.0000    Uiso 0.0127    4
C17   C    0.964(10)    0.2763(10)    1.0485(26)    1.0000    Uiso 0.0127    4
C21   C    0.870(14)    0.3483(12)    0.9903(28)    1.0000    Uiso 0.0127    4
C25   C    0.620(11)    0.4192(11)    0.8143(27)    1.0000    Uiso 0.0127    4
C29   C    0.638(13)    0.1180(12)    0.8334(30)    1.0000    Uiso 0.0127    4
```

H1	H	0.518(11)	0.2627(18)	0.7455(30)	1.0000	Uiso 0.0127	4
H5	H	0.989(12)	0.1482(15)	1.0452(27)	1.0000	Uiso 0.0127	4
H9	H	0.099(11)	0.2797(16)	1.1328(29)	1.0000	Uiso 0.0127	4
H13	H	0.941(11)	0.4083(16)	1.0260(31)	1.0000	Uiso 0.0127	4
H17	H	0.681(14)	0.5389(17)	0.816(4)	1.0000	Uiso 0.0127	4
H21	H	0.661(11)	1.0014(18)	0.857(4)	1.0000	Uiso 0.0127	4
O1	O	0.731(11)	0.4872(16)	0.8676(30)	1.0000	Uiso 0.0127	4
O5	O	0.461(13)	0.4171(22)	0.717(3)	1.0000	Uiso 0.0127	4
O9	O	0.447(13)	0.1145(21)	0.742(4)	1.0000	Uiso 0.0127	4
O13	O	0.702(12)	0.0546(16)	0.902(4)	1.0000	Uiso 0.0127	4

```
loop_   _atom_type_symbol _atom_type_number_in_cell
   C          32
   H          24
   O          16

# Note that Z affects _cell_formula_sum and _weight
_cell_formula_units_Z    4
_chemical_formula_sum    "C8 H6 O4"
_chemical_formula_weight    172.17
```

Pb Fcc EOS data points

a Pb (Å)	volume (Å^3)	Pressure (GPa)
4.96330	122.268	1 atm
4.90930	118.320	1.5
4.84392	114.361	3.4
4.75302	107.377	7.7
4.67040	101.874	12.3
4.61168	98.079	16.3
4.56770	95.300	19.8

References

1. Zheng, X. et al. Nearly unity quantum yield persistent room-temperature phosphorescence from heavy atom-free rigid inorganic/organic hybrid frameworks. *Angew. Chem. Int. Ed* **61**, e202207104 (2022).
2. Dewaele, A. et al. Stability and equation of state of face-centered cubic and hexagonal close packed phases of argon under pressure. *Sci. Rep.* **11** (2021).
3. Yamamoto, M., Oshima, K. & Matsubara, S. Platinum catalyzed HD exchange reaction of various aromatic compounds under hydrothermal condition. *Heterocycles* **67** (2006).
4. Matsubara, S., Yokota, Y. & Oshima, K. Palladium-Catalyzed H–D Exchange Reaction under Hydrothermal Condition. *Chem. Lett.* **33**, 294-295 (2004).
5. Zhang, Z. Y. et al. A synergistic enhancement strategy for realizing ultralong and efficient room-temperature phosphorescence. *Angew. Chem. Int. Ed.* **59**, 18748-18754 (2020).
