# Supplementary material for: Brightening triplet excitons enable high-performance white-light emission in organic small molecules via integrating n–π*/π–π* transitions
Source: Nat Commun. 2024 Sep 5;15:7778. doi: 10.1038/s41467-024-52196-7 (PMC11377787; doi:10.1038/s41467-024-52196-7)
Supplement: Supplementary file 2 — Description of Additional Supplementary Files [file 41467_2024_52196_MOESM2_ESM.pdf]

Description of Additional Supplementary Files

File Name: Supplementary Movie
Description: The phosphorescence of IPA before and after pressure treatment.
